# Born of frustration: the emergence of *Camelina sativa* as a platform for lipid biotechnology

**DOI:** 10.1093/plphys/kiaf009

**Published:** 2025-01-15

**Authors:** Richard P Haslam, Louise V Michaelson, Peter J Eastmond, Johnathan A Napier

**Affiliations:** Rothamsted Research, West Common, Harpenden AL5 2JQ, UK; Rothamsted Research, West Common, Harpenden AL5 2JQ, UK; Rothamsted Research, West Common, Harpenden AL5 2JQ, UK; Rothamsted Research, West Common, Harpenden AL5 2JQ, UK

## Abstract

The emerging crop *Camelina sativa* (L.) Crantz (camelina) is a Brassicaceae oilseed with a rapidly growing reputation for the deployment of advanced lipid biotechnology and metabolic engineering. Camelina is recognized by agronomists for its traits, including yield, oil/protein content, drought tolerance, limited input requirements, plasticity, and resilience. Its utility as a platform for metabolic engineering was then quickly recognized, and biotechnologists have benefited from its short life cycle and facile genetic transformation, producing numerous transgenic interventions to modify seed lipid content and generate novel products. The desire to work with a plant that is both a model and crop has driven the expansion of research resources for camelina, including increased availability of genome and other -omics data sets. Collectively, the expansion of these resources has established camelina as an ideal plant to study the regulation of lipid metabolism and genetic improvement. Furthermore, the unique characteristics of camelina enables the design-build-test-learn cycle to be transitioned from the controlled environment to the field. Complex metabolic engineering to synthesize and accumulate high levels of novel fatty acids and modified oils in seeds can be deployed, tested, and undergo rounds of iteration in agronomically relevant environments. Engineered camelina oils are now increasingly being developed and used to sustainably supply improved nutrition, feed, biofuels, and fossil fuel replacements for high-value chemical products. In this review, we provide a summary of seed fatty acid synthesis and oil assembly in camelina, highlighting how discovery research in camelina supports the advance of metabolic engineering toward the predictive manipulation of metabolism to produce desirable bio-based products. Further examples of innovation in camelina seed lipid engineering and crop improvement are then provided, describing how technologies (e.g. genetic modification [GM], gene editing [GE], RNAi, alongside GM and GE stacking) can be applied to produce new products and denude undesirable traits. Focusing on the production of long chain polyunsaturated omega-3 fatty acids in camelina, we describe how lipid biotechnology can transition from discovery to a commercial prototype. The prospects to produce structured triacylglycerol with fatty acids in specified stereospecific positions are also discussed, alongside the future outlook for the agronomic uptake of camelina lipid biotechnology.

## Introduction

Over recent years the annual oilseed crop *Camelina sativa* L. Crantz (camelina) has received renewed interest from multiple communities, including researchers, growers, processors, and policymakers. Each of these groups is attracted by the potential of camelina to address the challenges of agricultural sustainability, sourcing renewable biofuels and bio-based materials, and increasing climate resilience in our cropping systems. Camelina is a member of the Brassicaceae, native to Europe and found on farms across a huge range of geographical locations (e.g. Alaska to Argentina; Weiss et al. 2024). Recent reviews have captured the potential of camelina, which has (1) remarkable agronomic versatility, metabolic plasticity, and environmental adaptability; (2) low-input requirements; (3) resistance to pests and diseases; and (4) multiple uses in food, feed, and bio-based applications (Berti et al. 2016; Boutet et al. 2022; Zanetti et al. 2024). The agronomic adaptability of camelina (both spring and winter biotypes have been identified) has made it an attractive crop for integration into farming systems as an intermediate/cover/cash crop via intercropping, double cropping, or use on marginal lands. It is important to recognize that camelina is an oilseed crop, with seed oil yields typically comparable with that of *Brassica juncea* and *Brassica rapa* and higher than that of soya bean. Camelina may yet not match the oil yield of canola, but the costs of production (camelina has lower fertilizer requirements) can be less than one-half that of *Brassica napus* (canola/rapeseed). Camelina can therefore provide a route to profitability for agricultural regions with limited economic opportunities (Zanetti et al. 2024), and efforts are now underway using conventional breeding programs to develop new camelina varieties with improved agronomic traits (e.g. Ghidoli et al. 2024). Typically, camelina seeds contain approximately 38% to 42% oil, predominantly in the form of triacylglycerol (TAG), which consists of 3 fatty acids esterified to a glycerol backbone. Camelina shows remarkable morphological plasticity (Zanetti et al. 2021), and any variations in camelina seed oil and fatty acid composition typically reflect genetics, environmental conditions, and genotype-by-environment interactions (Brock et al. 2020). Camelina oil has a nutritionally beneficial profile rich in unsaturated fatty acids (n-6/n-3 ratio 0.6), including oleic (18:1^Δ9^ [total carbons:desaturations], 14% to 16%), linoleic, (18:2^Δ9,12^ 15% to 23%), α-linolenic (18:3^Δ9,12,15^ 31% to 40%), and eicosenoic (20:1^Δ9^ 12% to 15%) acids. Other minor fatty acids include palmitic (C16:0), stearic (C18:0), and erucic (22:1^Δ13^) acids. The accumulation of erucic acid and glucosinolates (defensive secondary metabolites commonly found in *Brassica* species) in camelina seeds is relatively low but can be a challenge for the use of this oil in some food and feed applications. Addressing these issues had been a task for both breeders and metabolic engineers alike.

Following the publication of genomes for the camelina doubled haploid DH55 accession (Kagale et al. 2014; see updated version http://cruciferseq.ca) and the spring biotype “Suneson” (Fang et al. 2023; Brock et al. 2024), efforts to improve camelina have been underpinned by the increasing number and diversity of genomic resources available. Bird et al. (2024) recently sequenced, assembled, and annotated 12 complete, chromosome-scale genomes of camelina. Camelina genomics resources are part of the Brassica database BRAD (http://brassicadb.org), and they are accessible via the JGI Phytozome BAP project and camelina pangenome sequencing effort (https://phytozome-next.jgi.doe.gov/info/CsativaJoelle_v1_1). Furthermore, camelina gene expression data can be examined in several different ways. For example, the University of Toronto has developed an image expression browser (Kagale et al. 2016; http://bar.utoronto.ca/) for camelina, representing expression data from a large developmental set. In addition, the Camelina Genomic Resources (camelinagenome.org) contains embryo transcript data on protein and lipids. The Camelina Gene Regulation Database (Gomez-Cano et al. 2020; http://camregbase.org/) provides a resource for aspects related to camelina gene regulation, including tissue-specific gene expression visualization and gene coexpression analyses. The reanalysis of published gene expression data has identified evidence for genome dominance in camelina, with the third subgenome dominant over the other 2 (Mandáková et al. 2019; Chaudhary et al. 2020). However, this is now thought to be largely restricted to floral and fruit organs (Brock et al. 2024), which has significance for seed lipid-engineering approaches that depend on endogenous metabolism. Collectively, these resources make camelina an excellent chassis for biotechnology. Metabolic engineers can make use of its simple “floral dip” transformation protocol (Lu and Kang 2008) and short life cycle to make rapid progress from the laboratory to the field. More broadly, discoveries in camelina are often directly transferable to staple commodity oilseed crops, for example canola, and are highly informative for other dicot oilseeds, including soybean.

## Seed lipid metabolism in camelina

Seed oil biosynthesis requires a supply of carbon. When exposed to light, oil-producing green seeds convert maternally supplied sugars and amino acids into storage products (starch, oil, and protein). Seed oil TAGs are composed of fatty acids generated by combining 2-carbon acetyl groups derived from 3-carbon pyruvate and requires energy (ATP) and reducing power (NADPH and NADH). The decarboxylation of pyruvate to acetyl CoA results in 33% of the carbon being converted to CO_2_. Seeds also respire carbon through the tricarboxylic acid cycle to produce adenosine triphosphate and carbon skeletons for amino acids used in protein biosynthesis. CO_2_ produced in green seeds can be refixed by Rubisco. However, camelina has a low carbon conversion efficiency (32% to 40%; Carey et al. 2020) compared with other green oilseeds and is characterized by a highly active oxidative pentose phosphate pathway, which produces excessive NADPH (used in fatty acid synthesis or dissipated by alternative oxidase AOX1). Latterly, research in camelina has demonstrated a role for pod walls to fix CO_2_ photosynthetically and contribute to seed biomass, enable seed filling, and maximize the number of viable seeds. In the absence of leaves, photosynthesis in pod walls has the capacity to contribute ∼79% of seed biomass (Koley et al. 2022).

**Figure 1.**
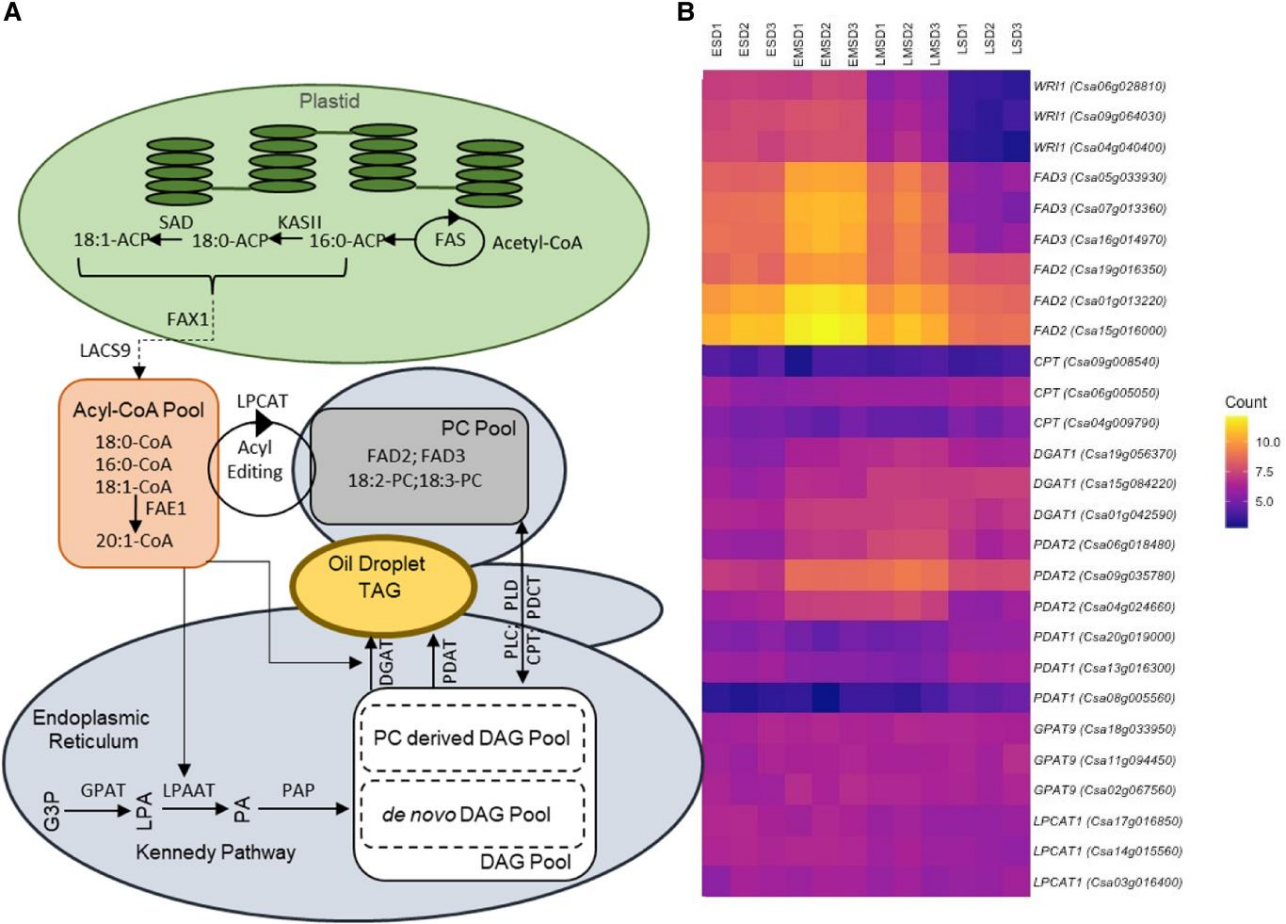
Camelina seed lipid biosynthesis. **A)** An illustration of endogenous cellular lipid synthesis and assembly pathways, including the following selected genes: FAS, fatty acid synthase; KASII, beta ketoacyl acyl carrier protein synthase II; SAD, stearoyl-acyl carrier protein desaturase; FAD, fatty acid desaturase; FAX1, fatty acid export 1; LACS9, long chain acyl—CoA synthetase 9; FAE1, fatty acid elongation 1; LPCAT, lysophosphatidylcholine acyltransferase; DGAT, diacylglycerol acyltransferase; PDAT, phospholipid:diacylglycerol acyltransferase; PLC, phospholipase C; PLD, phospholipase D; CPT, diacylglycerol cholinephosphotransferase; PDCT, phosphatidylcholine diacylglycerol cholinephosphotransferase; GPAT, Glycerol-3-phosphate acyltransferase; LPAAT, lysophosphatidyl acyltransferase; PAP, phosphatidate phosphatase. **B)** Heatmap showing the expression of selected *C. sativa* genes during seed development generated using https://github.com/richysix/bioinf-gen/blob/master/docs/gene_expr_heatmap/gene_expr_heatmap.md (B). The color scale represents normalized counts calculated by DESeq2. RNA sequencing reads generated by Kagale et al. (2016) (accessions SRX472942, SRX472943, SRX472945, and SRX472946) were aligned to the *C. sativa* reference genome (Kagale et al. 2014) (accession JFZQ00000000) using TopHat and quantified at the gene level with HTSeq. Key to seed stages: Early seed development (ESD), Early mid seed development (EMSD), Late mid seed development (LMSD), and Late seed development (LSD).

Camelina shares many features of fatty acid synthesis with other oilseeds, and the complexities of lipid synthesis and oil assembly have been reviewed elsewhere (see Li-Beisson et al. 2013; Bates 2016; Yang et al. 2022; camelina pathway descriptions in Xu et al. 2024; www.fatplants.net/). Briefly, fatty acids are generated in the plastid, whereas TAG assembly occurs outside the plastid in the endoplasmic reticulum (ER) and the TAG is packaged into oil bodies (see Fig. 1A for summary). The first committed step in the pathway is catalyzed by plastidial acetyl-CoA carboxylase (ACCase). Assembly of fatty acids occurs on an acyl carrier protein (ACP) via a cycle of reactions that elongate the carbon chain by 2 carbons. After 7 cycles, the saturated 16 carbon acyl-ACP can either be hydrolyzed by the FATB acyl-ACP thioesterase or further elongated by beta-ketoacyl-ACP synthase II to 18:0-ACP, which is then desaturated (Δ9–18:0-ACP desaturase) to 18:1-ACP and hydrolyzed by the FATA thioesterase to produce C16:0 and C18:1, with their relative proportions determined by the activities of FATA, FATB, Δ9–18:0-ACP desaturase, and beta-ketoacyl-ACP synthase II. Polyunsaturated fatty acids are synthesized by 1 of 2 parallel pathways commonly referred to as the prokaryotic and eukaryotic pathways, located in the plastid and ER, respectively. Fatty acids are esterified to different lipids in the 2 compartments, phosphatidycholine (PC) in the ER and glycosylglycerides in the plastid. Conversion of C18:1 to C18:2 is mediated by FATTY ACID DESATURASE 2 (FAD2) in the ER and by FAD6 in the plastids, and conversion of C18:2 to C18:3 is mediated by FAD3 in the ER and by either FAD7 or FAD8 in the plastid. The synthesis of very long chain fatty acids (VLCFA) such as C22:1 is by the FA elongation enzyme complex located at the ER membrane. The complex sequentially adds 2 carbon units to C18:1, growing the acyl chain using 4 core enzymes, namely multigene family 3-ketoacyl-CoA synthase (KCS), 3-ketoacyl-CoA reductase, 3-hydroxyacyl-CoA dehydratase, and trans-2,3-enoyl-CoA reductase. FATTY ACID ELONGASE1 (FAE1)-encoded β-ketoacyl-CoA synthase is a specific member of the KCS family and is a rate-limiting enzyme directing the elongation of C18:1; therefore, KCS/FAE1 is an important regulatory target for altering VLCFA content through metabolic engineering.

Overall production of fatty acids is regulated by both the transcription factor WRINKLED 1 (WRI1; Cernac and Benning 2004) and the complex biochemical network of control surrounding ACCase, for example, phosphorylation, redox status, PII interactions, and feedback regulation by 18:1-ACP (reviewed in Conrado et al. 2024). Several additional genes have been identified in Arabidopsis to regulate seed fatty acids synthesis and oil accumulation, including LEAFY COTYLEDON 2 (LEC2), ABSCISIC ACID INSENSITIVE 3 (ABI3), and FUSCA3 (FUS3) (Miray et al. 2021). However, aspects of seed lipid metabolism in camelina are unique, and Gomez-Cano et al. (2022) identified CsaMYB1, CsaABI3AVP1-2, CsaHB1, CsaNAC2, CsaMYB3, and CsaNAC1 as regulators likely involved in the control of seed fatty acid elongation, and CsaABI3AVP1-2 and CsabZIP1 as potential regulators of the synthesis and degradation of TAGs. After export, long-chain acyl-CoA synthetase converts free fatty acids to acyl-CoA for the assembly of TAG. Glycerol-3-phosphate and acyl-CoAs are synthesized into TAG via the Kennedy pathway (Fig. 1A), a series of activities including 2 acylations of G3P by sn-1 glycerol-3-phosphate acyltransferase and lysophosphatidic acid acyltransferase, followed by phosphatidic acid phosphatase, and a third acylation by diacylglycerol acyltransferase (DGAT). Alternatively, newly synthesized FAs can enter the PC pool via acyl-CoA:lysophosphatidylcholine acyltransferase. PC plays a central role in TAG synthesis through acyl editing where further desaturation or modification can occur before release back into the acyl-CoA pool for participation in TAG synthesis, direct transfer of fatty acids to diacylglycerol (DAG) producing TAG via phospholipid:diacylglycerol acyltransferase (PDAT) and utilization of PC-derived DAG as a substrate for TAG synthesis. Analysis of camelina seed total DAG identified a predominance of linoleic and linolenic acids (e.g. 36:5 18:2/18:3, 36:6 18:3/18:3, and 36:4 18:2/18:2; 55% of total DAG), alongside other DAG molecular species containing VLCFA, such as 38:4 (20:1/18:3) and 38:3 (20:1/18:2) at 11% and 7%, respectively (Rodríguez-Rodríguez et al. 2021). Studies of TAG biosynthesis kinetics using [^14^C]glycerol labeling demonstrated a role for the PC-derived DAG pathway in camelina but identified a significantly greater flux through the Kennedy pathway relative to other oilseeds such as Arabidopsis or soybean (Bates et al. 2014).

In developing camelina embryos, the maximum rate of oil synthesis is at mid-maturation, that is, between 14 and 20 days post-anthesis (DPA), while the mid-point for oil deposition is around 17 to 18 DPA. A striking feature of seed maturation in camelina is the late-stage surge and then abrupt cessation in C18:3 deposition. The rate of C18:1 deposition also dips during mid-maturation, consistent with its role as precursor for C18:2, C18:3, and C20:1 biosynthesis. Synthesized by FAE1, C20:1 is first detected at 11 DPA and the production of other minor VLCFAs, such as eicosanoic acid (C20:0) and erucic acid (C22:1), closely parallels that of C20:1. Also, in late seed maturation, C18:0 accumulation is greatly reduced relative to C16:0 (see detailed description provided in Pollard et al. 2015 and developmental changes in lipid biosynthesis transcripts illustrated in Fig. 1B). Camelina seed TAG fatty acid composition characteristically reflects the total lipid profile and electrospray ionisation mass spectrometry analysis identifies abundant 52:3—52:6, 54:2—54:9, 56:2—56:9, and 58:2—58:7 molecular species; alongside some minor 60:x, 62:x, and 64:x species. The leaf fatty acid 16:3 and odd chain fatty acids are rarely found in seed TAG. Seed TAG content and composition are not fixed; indeed, a small family of TAG lipase genes in Brassicas (Eastmond 2006), consisting of SUGAR-DEPENDENT1 (SDP1) and SDP1-LIKE (SDP1L), is responsible for a decline in seed oil content (∼10%) in maturing oilseeds. The suppression of SDP1 in *Brassica napus* during seed development resulted in an 8% gain in seed oil yield and is recognized by researchers as a route to enhance oil yield in seeds (Kelly et al. 2013). A further TAG lipase (TAGL) has been identified (Horn et al. 2016) in seeds from the Brassicaceae *Physaria fendleri*, and its upregulation during seed development in camelina engineered to produce novel hydroxy fatty acids has been observed. Lipase (TAGL) activity has now been shown to have a role in TAG remodeling, enabling the accumulation of unusual fatty acids in multiple positions in the glycerol backbone of TAG and demonstrating that TAG should be considered not as a metabolic endpoint but a dynamic pool (Bhandari and Bates 2021; Parchuri et al. 2024), as previously proposed in the 1990s.

**Figure 2.**
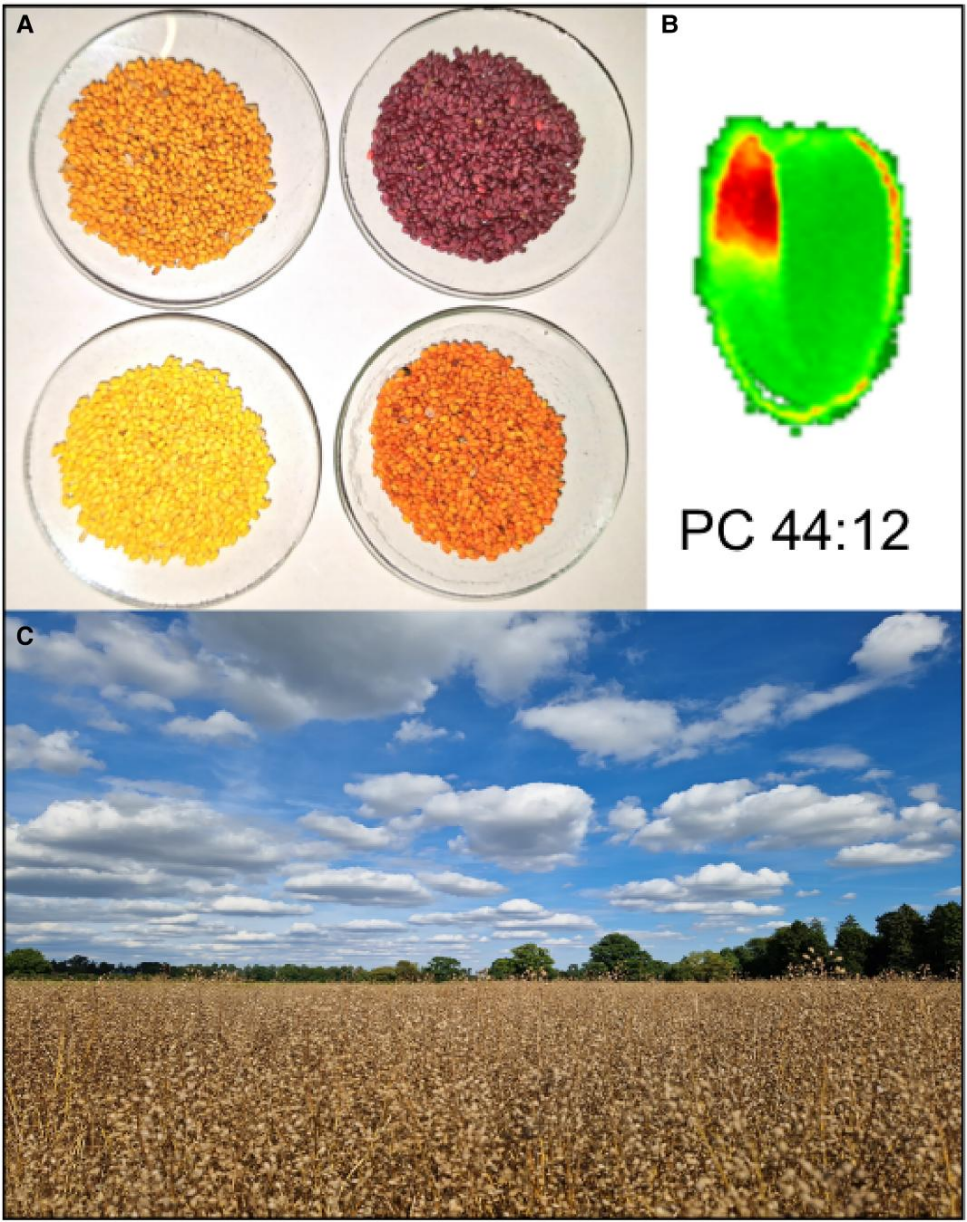
Camelina as a platform of metabolic engineering. **A)** Field-grown camelina seed engineered with a range of traits including omega-3 and ketocarotenoids (seeds from modified camelina are shown clockwise) 1. Wildtype; 2. RUBY (betaline); 3. Astaxanthin; and 4. tt2 mutant (MYB); **B)** Representative MSI study (camelina seed section; coloring indicates abundance—red high and green low) highlighting the asymmetric accumulation (in the embryonic axis tip) of a selected PC molecular species containing novel fatty acids (C22:6/C22:6; see Usher et al. 2017); **C)** Field testing (Rothamsted Research, UK) of Camelina sativa engineered for the production of omega-3 long chain polyunsaturated fatty acids (EPA and DHA).

Aside from TAG, other lipid species contribute to the camelina seed total lipids. This includes contributions from plastid localized lipids, including monoglalactosyldiacylglycerol, phosphatidylglycerol, diglalactosyldiacylglycerol, and sulfoquinovosyldiacylglycerol. The synthesis of these plastid lipids peaks at approximately 16 DPA and then continues to decline through seed maturity. The other major membrane glycerolipids are PC, phosphatidylethanolamine, and phosphatidylinositol. The PC pool is central to seed lipid metabolism and oil synthesis; it provides substrates for desaturation, acyl editing, and TAG synthesis. However, the composition of PC is dynamic and changes during seed maturation, reflecting the production of C18:3 and C20:1 relative to C18:1 and C18:2. Analysis of seeds from the Brassicaceae, including camelina, has shown that the embryo typically provides 85% to 90% of the seed oil content (Li et al. 2006), but the embryo has a complexity of cell types and differentiated lipid metabolism. Over recent years, this complexity has been addressed using mass spectrometry imaging of seed sections to provide a detailed analysis of TAG (product) and PC (precursor) tissue-specific lipid distributions (Horn and Chapman 2024). This approach identified unexpected heterogeneity in camelina seed lipid distributions, with PC and TAG species enriched in C18:2 preferentially localized to the embryonic axis and lipid classes enriched in C20:1 preferentially localized to the cotyledons (Horn et al. 2013). This asymmetric distribution has identified the potential for tissue-specific biosynthetic pathways (Fig. 2B). TAG production in seeds reflects the precursor specificity of the acyltransferases PDAT and DGAT; DGAT has a higher selectivity for C20:1, and PDAT is more likely to incorporate C18:2 into TAG. The lack of tri-18:2 in the cotyledon suggests DGAT predominates in cotyledons and PDAT is more significant in the embryonic axis (a distribution supported by a PDAT and DGAT knockdown study in camelina; Marmon et al. 2017). The further identification of PC species containing C18:3 in the outer cotyledon indicated not only a developmental regulation of FAD3 but also the possibility of tissue-specific expression.

## Demonstrating the efficacy of camelina as a platform for metabolic engineering

Historically, manipulation of camelina seed for oil content and composition has included identifying natural (Vollmann et al. 2007) and induced (ethyl methanesulfonate; e.g. Büchsenschütz-Nothdurft et al. 1998) variation. The key target genes for manipulation have included genes for fatty acid desaturases that control polyunsaturated fatty acid production, for example, FAD2 that forms C18:2 by Δ12 desaturation, FAD3 for C18:3 production, and FAE1 that elongates C18:1 to C20:1 and C22:1, respectively. By screening ethyl methanesulfonate camelina lines, Neumann et al. (2021) were able to identify mutants in *FAE1*, *FAD2*, and *FAD3* and successfully stack these traits into 1 line to produce a camelina line with mid-oleic acid oil. However, opportunities for innovation have prompted the application of biotechnological strategies for trait improvement in camelina. Predominantly many of these approaches have focused on seed traits, including yield (seed size and number), oil content, and composition (Supplementary Table S1). Metabolic engineering of these traits can be a challenge given the complexity of endogenous seed lipid metabolism (discussed above; Fig. 1) and the polyploid genome of camelina with multiple copies of each gene (1 for each subgenome). Overcoming these challenges, researchers have successfully demonstrated how metabolic engineering can be used to improve oilseed crops and create sustainable solutions. The examples of metabolic engineering detailed in Supplementary Table S1 are not exhaustive but provide demonstrations of how biotechnology has been applied for crop improvement in camelina. Examples include the utilization of different approaches (RNA interference [RNAi], GE, and GM) and interventions to change seed oil composition. This includes RNAi-mediated suppression of chlorophyll b assembly for improved photosynthesis and field performance (Friedland et al. 2019), the production of high-oleate lines generated by RNAi-mediated suppression of *FAD2* and *FAE1*, producing high-linoleate lines (C18:2), effectively reducing the production of C18:3 and C20:1 in the seed (Horn et al. 2013; Nguyen et al. 2013). Reducing the production of VLCFA, including the undesirable erucic acid (C22:1), and increasing the production of 18 carbon fatty acids has led to metabolic engineers targeting *FAE1* using RNAi (Bashiri et al. 2023) and GE methods (Ozseyhan et al. 2018).

GE has been extensively applied in camelina for crop improvement. For example, to reduce FAE1 expression and lower seed VLCFAs (60% reduction; Ozseyhan et al. 2018), generate FAD2 knock outs with decreased 18:2 and 18:3 producing seeds with increased MUFA content (Jiang et al. 2017; Lee et al. 2021), compositional changes resulting from the knock down of DGAT1 and 2 (Lee et al. 2024), and disruption of the Transparent Testa 8 transcription factor, increasing seed total fatty acid, TAG content, and producing heavier seed weights (Cai et al. 2024). Furthermore, efficient multiplex GE has been demonstrated in the hexaploid camelina (see Bellec et al. 2022), producing early-flowering biotypes suitable for summer cropping by targeting flowering repressor genes (FLOWERING LOCUS C, SHORT VEGETATIVE PHASE, LIKE HETEROCHROMATIN PROTEIN 1, TERMINAL FLOWER 1 and EARLY FLOWERING LOCUS 3). Titration of the induced combinatorial mutations identified early-flowering phenotypes stable for 5 generations. Other iterations include the use of GE to address potential adverse properties of camelina seeds, for example, glucosinolates, that can represent a limitation on the use of Brassica species in livestock feed. Cas9 endonuclease-based targeted mutagenesis of the glucosinolate transporters CsGTR1 and CsGTR2 caused a strong decrease in glucosinolate amounts, while mutagenesis of each glucosinolate biosynthesis transcription factor (CsMYB28 and CsMYB29) homeolog resulted in the complete loss of glucosinolates, representing the first glucosinolate-free Brassicaceae crop (Hölzl et al. 2023). As illustrated in Supplementary Table S1, increasing seed oil content has been addressed by several different approaches to manipulate seed fatty acid production and lipid assembly pathways, including the expression of the transcription factor WRI1 (An and Suh 2015); fatty acid transporters FAX1 and members of the ATP-binding cassette transporter subfamily A9 (Cai et al. 2021); oil biosynthesis enzymes PDAT (Abdullah et al. 2024) and DGAT1 (Kim et al. 2016); DGAT1 and a yeast cytosolic glycerol-3-phosphate dehydrogenase (Chhikara et al. 2018); overexpression of the ACCase subunit α-CT (Wang et al. 2022); vacuolar sugar transporter TST1 (Okooboh et al. 2022); and fatty acid exchange via a nonspecific phospholipase C6 (Cai et al. 2020). Improving the performance (flower fertility, yield, oil content, and plant architecture) of camelina has also received attention through expression of CYP78A genes of the P450 monooxygenase family previously demonstrated to be involved in regulating seed development in Arabidopsis [AtCYP78A6 or AtCYP78A5; Hölzl and Dörmann (2021)] and via improved CO_2_ use efficiency [expression of the *E. coli* chloroplast glycolate dehydrogenase, glyoxylate carboxylase, and tartronic semialdehyde reductase; Dalal et al. (2015)] enhancing growth of transgenic camelina plants, with larger capsules and seeds. These are just some highlighted examples of biotechnological crop improvement in camelina; additional examples are described by Yu et al. (2018).

The most striking examples of biotechnology for crop improvement in camelina occur when GM approaches are used to redesign seed metabolism to produce higher value food and nonfood products. A demonstration of this is the diversion of sinapine precursors to produce a value-added co-product such as 4-vinyl phenols. Sinanpine is an antinutritional compound found in Brassica species that reduces the suitability of protein-rich seed meal for use in animal feed, while 4-vinyl phenols have utility in a range of industrial applications. Rather than using a genetic intervention to reduce sinapine levels, Menard et al. (2022) expressed a modified bacterial phenolic acid decarboxylase in developing camelina to redirect phenylpropanoid pathway flux from sinapine biosynthesis to the production of 4-vinyl phenols, providing a non-petrochemical source of this class of industrial chemicals. Metabolic engineers have successfully utilized camelina for the sustainable production of many valuable biobased products (reviewed in Bansal and Durrett 2016; Yuan and Li 2020; Ghidoli et al. 2023). Several demonstrations of these approaches are provided in Supplementary Table S1 and include the production, via metabolic engineering, of novel fatty acids, including hydroxyl fatty acids (Snapp et al. 2014; Aryal and Lu 2018), 3-acetyl-1,2-diacyl-sn-glycerols with medium-chain fatty acids (Liu et al. 2015; Bansal et al. 2018; Alkotami et al. 2024), production of nervonic acid (C24:1 Δ15; Huai et al. 2015), ω-7 fatty acids (Nguyen et al. 2015; Rodríguez-Rodríguez et al. 2021), precursors for sustainable aviation fuel including capric and myristic fatty acid production (Kim et al. 2015), and cyclopropane fatty acid accumulation (Yu et al. 2019). Metabolic engineering in camelina has further validated routes to the production of industrial lubricants (waxes; Iven et al. 2016; Zhu et al. 2016; Ruiz-Lopez et al. 2017) and replacements for the chemical feedstocks used to create plastics and polymers (Malik et al. 2015, 2023). These approaches support the transition from our dependence on fossil fuels, providing a biobased alternative in an existing oilseed cropping system.

## Transition from proof of concept to prototype: nutritional enhancement of camelina

The application of biotechnological approaches in camelina has enabled researchers to develop metabolic engineering strategics to improve the nutritional quality of oilseeds (e.g. increased production of tocohromanols; Konda et al. 2023, and carotenoids; He et al. 2022) and produce new supplies of finite resources. This is particularly true for aquaculture, which relies on marine extraction for key feed ingredients, including omega-3 long chain polyunsaturated fatty acids [omega-3 LC-PUFAs; eicosapentaenoic acid (EPA; 20:5^Δ5,8,11,14,17^) and docosahexaenoic acid (DHA; 22:6^Δ4,7,10,13,16,19^)], which are present only in marine food webs. Healthy diets depend on key nutrients such as omega-3 LC-PUFAs, and their sustainable supply is a challenge for our existing food systems. The production of these novel fatty acids in oilseeds is not straightforward and required the reconstruction and coordination of a complex seed-specific biosynthetic pathway in camelina comprising a series of enzymatic reactions that convert endogenous C18 fatty acids to C20+ LC-PUFAs (reviewed in Napier and Betancor 2023; Venegas-Calerón and Napier 2023). The successful reconstitution of this pathway involved extensive iteration to define the optimal gene combinations that effectively combined with endogenous camelina seed lipid metabolism to produce a terrestrial seed oil with EPA and DHA levels matching those found in commercial fish oil (Ruiz-Lopez et al. 2013; Ruiz-Lopez et al. 2014). Using the same approach, a tailored camelina oil was generated accumulating EPA and the related omega-3 LC-PUFA eicosatetraenoic acid (20:4^Δ8,11,14,17^) (Ruiz-Lopez et al. 2015). Seed oils such as these represented an additional source of EPA and an entirely new source of the bona fide fish oil eicosatetraenoic acid. At this point, camelina EPA + DHA oil was a proof of concept (Fig. 2A and C), like many of the demonstrations illustrated in Supplementary Table S1. To achieve the successful translation of this biotechnology to a prototype required further validation steps. First, oils derived from omega-3 (EPA + DHA)-rich camelina were successfully trialed as substitutes for fish oil in feed diets used in aquaculture. The trials showed that fish fed the GM-derived oils had enhanced levels of n-3 LC-PUFA in their flesh compared with either a commercial diet control or a feed containing wild-type camelina. Indeed, in some cases, salmon fed a diet containing high omega-3 LC-PUFA camelina oil accumulated almost double the amount of these health beneficial fatty acids as salmon fed a fish oil diet (Betancor et al. 2017; Napier et al. 2020). Secondly, the stability of the camelina seed EPA + DHA trait had to be confirmed in field situations, effectively incorporating field trialing into the engineering biology “design-build-test-learn” cycle. Therefore, the transgene-directed accumulation of non-native omega-3 long chain polyunsaturated fatty acids in the seed oil of camelina was evaluated in distinct geographical and regulatory locations (UK, USA, and Canada; Han et al. 2020). The accumulation of EPA + DHA in seeds was found to be consistent with controlled environment experiments irrespective of the agricultural environment, demonstrating the stability and robust nature of the omega-3 trait (and camelina as a host for enhanced lipid metabolism). Additional examination of field grown nonseed tissues for the unintended accumulation of EPA and DHA failed to identify their presence, further confirming the seed-specific accumulation of these novel fatty acids (Han et al. 2022b). The asymmetric distribution of camelina oil assembly pathways noted in earlier sections (predominant DGAT activity in cotyledons and significant PDAT activity within the embryonic axis) impacted EPA + DHA biosynthesis and accumulation in seeds; MSI approaches found that novel fatty acids preferentially accumulated in the embryonic axis of glasshouse and field grown material (Usher et al. 2017), demonstrating how tissue-specific endogenous oil biosynthesis influences the accumulation of novel fatty acids in seeds (Fig. 2B). Combining transgene-derived seed-specific synthesis of omega-3 LC-PUFAs with CRISPR-Cas9 GE to inactivate the FAE1 pathway in a “GM + GE stack” and evaluating this iteration in a field environment further demonstrated that the accumulation of EPA + DHA can be increased by augmenting the α-linolenic acid precursor pool and shutting off the carbon shunt into 20:1^Δ11^ and 22:1^Δ13^ (Han et al. 2022a) Finally, if omega-3 (EPA + DHA) camelina oil is to be a means of provisioning the human population with an alternative supply of EPA and DHA, studies were required to test if EPA and DHA consumed as oil from transgenic camelina are incorporated after a meal into blood lipids at least as well as when consumed as fish oil. A double blind, postprandial cross-over trial concluded that there were no significant differences between test oils or sexes in EPA and DHA incorporation into plasma TAG and PC over the duration of the trial. The incorporation into blood lipids of EPA and DHA consumed as camelina EPA + DHA oil was equivalent to commercial blended fish oil, and the efficacy of transgenic camelina derived oils as a suitable dietary source of EPA and DHA in humans was demonstrated (West et al. 2019; West et al. 2020). Producing omega-3 LC-PUFAs in camelina seeds and validating the derived oils confirmed the stability and utility of this oil in both indirect and direct human nutrition, effectively derisked the biotechnology and provided the impetus for its commercialization via public-private partnership. Collectively these discovery and development efforts have established a road map for the translation of camelina biotechnology. The production of EPA + DHA in camelina and other oilseeds such as canola (Event NS-B50027-4; Petrie et al. 2020; Event BPS-BFLFK-2; Andre et al. 2019) has been a huge effort across the lipid community and represents some of the most complex engineering in plants to date.

## Prospects for engineering new types of nutritional lipids in camelina seed TAG

The successful development of oils rich in omega-3 LC-PUFAs provides a road map for engineering production of other dietary lipids in camelina, such as sustainable alternatives to animal fats. The TAGs found in meat and milk differ from those of plants in both the types of FAs that are esterified to the glycerol backbone and their stereospecific positions (sn-1, 2, or 3) (Michalski et al. 2013). These differences not only affect the physicochemical properties of the TAGs but also their digestion, absorption, and metabolic fate (Michalski et al. 2013). For example, more than 70% of the C16:0 found in human milk fat is esterified to the sn-2 position on the glycerol backbone, and 1,3-dioleoyl-2-palmitoylglycerol is the predominant TAG species (Wei et al. 2019). Harboring C16:0 in this specific TAG stereoisomer is known to be beneficial for lipid and calcium absorption in the infant gut (Béghin et al. 2018). Dietary TAGs are hydrolyzed in the duodenum by pancreatic lipase, which is sn-1/3 specific. The 2-monoacylglycerol and free fatty acids that are released are absorbed by intestinal mucosal cells. However, saturated long chain fatty acids, such as C16:0, are absorbed less efficiently than unsaturated fatty acids because they form insoluble soaps with calcium and magnesium ions, whereas 2-monoacylglycerols are absorbed well regardless of their fatty acyl group (Wei et al. 2019). Vegetable fats are used in most infant formulas, but plants only esterify C16:0 to the sn-1/3 position and not to sn-2 (van Erp et al. 2019). *Arabidopsis thaliana* seeds have been engineered to alter the stereospecific position of C16:0 to sn-2 (van Erp et al. 2019) and ultimately to produce TAG with a similar level of 1,3-dioleoyl-2-palmitoylglycerol to human milk fat (van Erp et al. 2021). This engineering strategy could potentially be applied to camelina. It is noteworthy that animal fats generally harbor considerably more C16:0 in the sn-2 position than do plant fats (Michalski et al. 2013). Dairy milk fat additionally contains a range of short and medium chain saturated FA, with short chain, predominantly butyrate (C4:0), found only at the sn-3 position (Michalski et al. 2013).

## Conclusion and perspectives

The renaissance of camelina over recent years reflects its potential contribution to some of society's most significant challenges, including nutritional security and the supply of sustainable feedstocks and products, all enshrined in the UN Sustainable Development Goals. The challenge of food production in a changing climate, alongside the transition from fossil fuels, is driving research and innovation in nonstaple crops like camelina. As illustrated here, the successful exploration and application of metabolic engineering can provide solutions. Although the solutions described above may have associated regulatory burdens, they have the advantage of utilizing the preexisting infrastructure and know-how that underpin modern agriculture, allowing rapid scaling and incorporation into production cycles. The future of camelina will involve continued iterative advancement, developing new traits such as improved protein content and composition (Supplementary Table S1) and stacking trait combinations together to develop new biotypes that incorporate climate-resilience and novel products. Expanding our use of plant-derived feed and foodstuffs will be essential for operating within planetary boundaries (Willett et al. 2019), and collectively we need to embrace a different approach—enabling plant biotechnology to play a key role by supporting both the discovery *and validation* of metabolic engineering approaches.

**Advances Box**

- **Breakthrough techniques for lipid analysis:** The advent of affordable benchtop mass spectrometry has enabled researchers to develop techniques (e.g. targeted and untargeted liquid chromatography mass spectrometry, LC-MS, direct infusion; hydrophobic interaction liquid chromatography, HILIC; multi reaction monitoring, MRM; and high-resolution accurate mass, HR/AM), for the quantitative assessment of individual lipid classes and molecular species (plant lipidomics). These approaches, along with radiolabelled flux analysis, have enabled the complete characterization of seed lipid pools and the exchange of fatty acids between lipid classes. In turn, this has permitted a greater understanding of complex lipid assembly and the interaction of endogenous metabolism with novel activities (Bates et al. 2014).
- **Spatially resolved seed lipid metabolism:** tissue-specific transcriptomics, proteomics and lipidomics (combined analysis in multi-omic experiments) has revised our (whole seed) understanding of seed lipid metabolism and its distribution of activities. Mass spectrometry imaging has provided striking images of asymmetric lipid molecular species distributions within seeds.
- **Iterative metabolic engineering and the design-build-test-learn cycle:** the straightforward reconstitution of novel metabolic pathways in seeds is rarely simple and requires the careful optimisation of gene candidates and combinations before the accumulation of desired products is achieved. Importantly, some plant metabolic engineers are now incorporating field testing into the DBTL-cycle to ensure desired outcomes are stable in real world environments.
- **The utility of plant biotechnology to deliver to sustainability goals:** by definition, plant biotechnology involves the use of genetic modification and/or gene editing, Approaches that require researchers to secure regulatory, funding, and societal acceptance. However, despite these hurdles, plant biotechnology provides a significant opportunity to mitigate the impacts of climate change, transition away from fossil fuels and support nutritional security.

**Outstanding Questions Box**

- How do we successfully design constructs, integrating regulatory elements and multiple transgenes, to ensure strong stable tissue-specific expression?
- What determines the stability of transgene expression across multiple generations?
- What causes the yield drag (reduced seed oil content) often associated with seed lipid metabolic engineering?
- Can we use ML/AI to improve our metabolic engineering, including predicting how modified plants will perform in the environment?
- How do we increase genetic diversity in camelina and optimise camelina ideotypes to specific pedoclimates?

## Supplementary Material

kiaf009_Supplementary_Data

## Data Availability

No new data were generated or analysed in support of this update.

## Supplementary material

The following materials are available in the online version of this article.

**Supplementary Table S1. Metabolic engineering in camelina.** Crop improvement and the deployment of novel traits in camelina using metabolic engineering approaches.

## Dive Curated Terms

The following phenotypic, genotypic, and functional terms are of significance to the work described in this paper:

SDP1 Gramene: AT5G04040
SDP1 Araport: AT5G04040

ABI3 Gramene: AT3G24650
ABI3 Araport: AT3G24650

FAD8 Gramene: AT5G05580
FAD8 Araport: AT5G05580

LEC2 Gramene: AT1G28300
LEC2 Araport: AT1G28300

seed AmiGo: PO:0009010

DGAT1 Gramene: AT2G19450
DGAT1 Araport: AT2G19450

FAD2 Gramene: NCBI GeneID: 820387
FAD2 Araport: NCBI GeneID: 820387

FATB Gramene: AT1G08510
FATB Araport: AT1G08510

FAD6 Gramene: AT4G30950
FAD6 Araport: AT4G30950

FAD7 Gramene: NCBI Gene ID: 544203
FAD7 Araport: NCBI Gene ID: 544203

WRI1 Gramene: AT3G54320
WRI1 Araport: AT3G54320

LACS9 Gramene: AT1G77590
LACS9 Araport: AT1G77590

FAX1 Gramene: At3g57280
FAX1 Araport: At3g57280

KASII Gramene: At1g74960
KASII Araport: At1g74960
