# Supplementary material for: Born of frustration: the emergence of Camelina sativa as a platform for lipid biotechnology
Source: Plant Physiol. 2025 Jan 15;197(2):kiaf009. doi: 10.1093/plphys/kiaf009 (PMC11812462; doi:10.1093/plphys/kiaf009)
Supplement: kiaf009_Supplementary_Data [file kiaf009_supplementary_data.docx]

| ***Trait*** | ***Intervention*** | ***Outcome*** | ***Reference*** |
| --- | --- | --- | --- |
| Seed oil content. | Expression of Arabidopsis patatin-related phospholipase pPLAIIIδ. | Increased seed oil content and decreasing cellulose content. | Li et al. (2015) |
|  | Overexpression of Camelina DGAT1B. | Total seed oils were increased by ~24%. | Kim et al. (2016) |
|  | Overexpression of *Pisum sativum* α-CT. | Average 8% increase in seed oil content. | Wang et al. (2022) |
|  | Overexpression of the Phosphatidylcholine: Diacylglycerol Cholinephosphotransferase. | Increased seed TAG content. | Abdullah et al. (2024) |
|  | Disruption of Transparent Testa 8. | Increased total fatty acid accumulation from 32.4% to as high as 38.0% of seed weight, and TAG yield by more than 21%. | Cai et al. (2024) |
| Seed oil composition. | RNAi suppression of Camelina *FAD2* and *FAE1*. | Increased accumulation of oleic acid. | Nguyen et al. (2013)  Horn et al. (2013) |
|  | Expression of microalgal and fungal genes for DHA synthesis. | Accumulation >12% of DHA, high ω3/ω6 ratio. | Petrie et al. (2014) |
|  | Expression of microalgal and fungal genes for EPA and DHA synthesis. | EPA and DHA content levels in camelina equivalent to those in fish oils. | Ruiz-Lopez et al. (2014) |
|  | Expression of *Ricinus communis* fatty acid hydroxylase (RcFAH), and Lesquerella condensing enzyme gene (LfKCS3). | High levels of hydroxyl fatty acid. | Snapp et al. (2014) |
|  | Expression of *Lunaria annua* Ketoacyl-CoA synthase (KCS) and three elongase genes from Arabidopsis. | Higher VLCFA production 6-12% (C24:1Δ15) nervonic acid. | Huai et al. (2015) |
|  | Different acyl-carrier-protein thioesterases (FATB) from *Caesalpinia pulcherrima*, *Cuphea viscosissima, Crocodylus palustris, Cladopus hookeriana* and *Umbellularia californica* and RNAi suppression of ketoacyl-ACP synthase II. | Medium chain FA of different lengths accumulation Capric, and myristic fatty acid production (jet fuel substrates). | Kim et al. (2015) |
|  | *Euonymus alatus* diacylglycerol-acetyltransferase (DAcT) overexpression with suppression of DGAT1 and/or PDAT1. | Modification and increased level of triacylglycerol content, and seed yield. | Liu et al. (2015) |
|  | Expression of microalgal and fungal genes for EPA and ETA synthesis. | Seed production of fish oil fatty acids EPA and ETA | Ruiz-Lopez et al. (2015) |
|  | *Umbellularia californica* lauryl-ACP thioesterase (FATB) expression and KASII suppression. | Accumulation of up to 40% of short chain laurate (C12:0) and myristate (C14:0) were present in seed TAG; suppression of KASII resulted in a further increase of palmitate, and reduction in longer, unsaturated fatty acids. | Hu et al. (2017) |
|  | CRISPR/Cas9 knock down of *FAD2*. | Decrease in seed linoleic and linolenic acid. | Jiang et al. (2017) |
|  | PDAT overexpression and DGAT suppression. | α-linolenic decrease and linoleic acid increase. | Marmon et al. (2017) |
|  | Co expression of phospholipase C-like protein (RcPLCL1) from castor bean and fatty acid hydroxylase (RcFAH12). | Production of hydroxy fatty acids. | Aryal et al. (2018) |
|  | Gene-edited FATTY ACID ELONGASE1 (FAE1) combined with the expression of EfDAcT, a high-activity diacylglycerol acetyltransferase isolated from *Euonymus fortune*, with suppression of the competing TAG-synthesizing enzyme DGAT1 | Ultra-high production of acetyl-TAGs. Production of 3-acetyl-1,2-diacyl-sn-glycerols (acetyl-TAGs) with medium-chain fatty acids (MCFAs). | Bansal et al. (2018) |
|  | CRISPR/Cas9 knock down of *FAE1*. | Over 60% reduction of seed VLCFAs. | Ozseyhan et al. (2018) |
|  | Over expression of microRNA167A. | Decreased alpha-linolenic acid content and increased seed size. | Na et al. (2019) |
|  | Expression of Lychee PHOSPHATIDYLCHOLINE:DIACYLGLYCEROL CHOLINEPHOSPHOTRANSFERASE (PDCT) & *Escherichia coli* CYCLOPROPANE SYNTHASE. | Cyclopropane fatty acid accumulation in seeds. | Yu et al (2019) |
|  | *CsFAD2* knockout plants via CRISPR-Cas9-mediated gene editing. | Enhanced MUFA levels (by 80%) in seeds. | Lee et al. (2021) |
|  | Crossing mutant lines to produce a fae1c/fad2a/fae1a/fad3a quadruple mutant. | Mid-oleic oils with nearly 40% oleic acid and reduced very long-chain (≤C20) fatty acid content. | Neumann et al. (2021) |
|  | RNAi for camelina β-ketoacyl-ACP synthase II and FAE1 combined with overexpression of FatA thioesterase and Δ9-acyl-ACP and acyl-CoA desaturases. | Enhanced production of ω-7 monounsaturated fatty acids. | Nguyen et al., 2015 |
|  | Expression of *Consolida ajacis* β-ketoacyl-ACP-synthase II like protein and *Arabidopsis thaliana* lysophosphatidic acid acyltransferase 2. | Increased synthesis of gondoic acid (cis-11 eicosenoic acid) and its positional redistribution to TAG sn-2. | Sarvas et al. (2021) |
|  | Iterative engineering for EPA and DHA synthesis and field testing. | Engineering EPA and DHA content levels in camelina equivalent to those in fish oils. | Han et al.  (2022a & 2022b) |
|  | Overexpression of a lysophosphatidic acid acyltransferase (LPAT). | Increased total phospholipid content, slight increase in seed size and accumulation of VLCFA. | Yin et al. (2022) |
|  | Expression of FAT5 16:0-CoA desaturase (*Caenorhabditis elegans*), and an engineered cyanobacterial 16:0/18:0-glycerolipid desaturase, DES9. | Reduced saturated fatty acids to 4.6%. | Bengtsson et al. (2023) |
|  | Antisense of FATTY ACID ELONGASE1 (FAE1) gene encoded the enzyme β-ketoacyl CoA synthase (KCS). | Reduction of erucic acid. | Bashiri et al. (2023) |
|  | Seed-specific expression of *Physaria fendleri* FAD3-1 (PfFAD3-1). | Up to 50% ALA content. | Park et al. (2023) |
|  | CRISPR/Cas9 knock down of DGAT1 and 2. | Small influence on the seed oil content and have an acyl preference for C20:1 and C18:3 | Lee et al. (2024) |
|  | *Agrobacterium tumefaciens* infiltration-based transformation of *C. neglecta*, overexpression of CvLPAT2, CpDGAT1 and CvFatB1 transgenes. | Medium chain seed fatty acid accumulation. | Wang et al. (2024) |
|  | Targeted engineering of camelina and pennycress seeds for ultrahigh accumulation of acetyl-TAG | Very high level accumulation of acetyl-TAGs | Alkotami et al. (2024) |
| Seed yield. | Expression of Arabidopsis G-protein γ subunit 3 (AGG3). | Increased seed size, number, and seed mass. | Choudhury et al. (2014) |
|  | Expression of Arabidopsis purple acid phosphate (AtPAP2). | Higher seed yields with increased seed size. | Zhang et al (2012) |
|  | *Expression of E.coli* chloroplast glycolate dehydrogenase (GDH), glyoxylate carboxylase (GCL), and tartronic semialdehyde reductase (TSR). | Enhanced CO_2_ use efficiency increased (up to 50%.) plant growth | Dalal et al. (2015) |
|  | Expression of chimeric Arabidopsis myosin XI-2. | Improved plant growth, total seed yield increase as the total seed number. | Duan et al. (2020) |
|  | Heterologous expression of a gene encoding a plastocyanin isoform. | Larger seeds with higher yield. Increased tolerance to salt stress. | Okooboh et al. (2023) |
| Seed Yield & Oil content. | Overexpression of Arabidopsis WRINKLED1 (AtWRI1). | Enhances seed oil content, seed mass and seed size. | An et al. (2015) |
|  | Expression of Arabidopsis diacylglycerol acyltransferase1 (DGAT1), and a yeast cytosolic glycerol-3-phosphate dehydrogenase (GPD1). | Up to 52% increase in seed mass, and up to 13% higher seed oil content. | Chhikara et al. (2018) |
|  | Expression of nonspecific phospholipase C6 (NPC6). | Increase seed oil content, seed weight, and oil yield. | Cai et al. (2020) |
|  | Expression of Arabidopsis lipid transporters, FAX1 (fatty acid export1), and ABCA9 (ATP-binding cassette transporter subfamily A9). | Increased expression of fatty acid, and seed oil production, increased seed weight and size. | Cai et al. (2021) |
|  | Overexpression/moderate expression of AtCYP78A6 or AtCYP78A5. | Expression levels impacted seed size/weight, oil content, and fertility. | Hölzl et al. (2021) |
|  | Overexpression of the vacuolar sugar transporter TST1. | Increased seed yield, oil content and root architecture. | Okooboh et al. (2022) |
| Plant growth. | Identification of EMS mutant camelina lines. | Resistance to acetolactate synthase inhibitor herbicides. | Walsh et al. (2012) |
|  | RNAi approach to reduce Chl b levels by modulating the expression levels of the chlorophyllide a oxygenase gene. | Reductions in Chl b levels and light harvesting antenna size. Increased photosynthesis and yield performance in the field. | Friedland et al. (2019) |
|  | Overexpression of WSD1. | Increased cuticle wax production and salinity tolerance. | Abdullah et al. (2021) |
|  | Multiplex editing of 15 target genes - FLOWERING LOCUS C, SHORT VEGETATIVE PHASE LIKE HETEROCHROMATIN PROTEIN 1, TERMINAL FLOWER 1 and EARLY FLOWERING LOCUS 3. | Identification of early flowering phenotypes; positive/negative impacts on yield. | Bellec et al. (2022) |
| Seed product. | Expressed fatty acyl-CoA reductase and wax ester synthase. | Production of wax esters in seeds. | Iven et al. (2016) |
|  | Co-expression of acyl-ACP thioesterases with *Marinobacter hydrocarbonoclasticus* WS and *Marinobacter aquaeolei* FAR. | Production of shorter chain novel wax ester species. | Ruiz-Lopez et al. (2017) |
|  | Overexpression of a non-native β-carotene ketolase gene coupled with other carotenoid biosynthesis genes (phytoene synthase, β-carotene hydroxylase, and Orange). | 70-fold increase in carotenoids. | He et al. (2022) |
|  | Expression of a modified bacterial phenolic acid decarboxylase (PAD). | 4-vinyl phenol glycosides production and reduced sinapine. | Menard et al. (2022) |
|  | Mutagenesis of glucosinolate transporters GTR1 and GTR2 and regulators of biosynthesis MYB28 and MYB29. | Reductions in seed glucosinolate content. | Hölzl et al. (2023) |
|  | Seed-specific HGGT expression with increased biosynthesis and/or reduced homogentisate catabolism. | Increased tocochromanol concentrations. | Konda et al. (2023) |
|  | Co-expression of plastid-targeted β-ketothiolase (PhaA), NADPH-dependent reductase (PhaB) and PHA synthase (PhaC) enzymes. | Polyhydroxybutyrate (10%) seed accumulation. | Malik et al. (2015)  Malik et al. (2023) |
|  | RNAi mediated suppression of seed ADP-glucose pyrophosphorylase (AGPase). | Enhanced seed protein content and seed size. | Na et al. (2018) |
| Seed protein. | CRISPR/Cas9 gene editing of seed storage protein CRUCIFERIN | Significant change in seed amino acid content; increase in the proportion of alanine, cysteine, and proline, and decrease of isoleucine, tyrosine and valine. | Lyzenga et al. (2019) |
|  | Expression of a feedback inhibition-insensitive form of dihydrodipicolinate synthase from *Corynebacterium glutamicums.* | Seed lysine content increased 13.6 -22.6%. | Huang et al. (2022) |
|  | Seed-specific RNAi lines deficient in napins were generated by targeting 2S SSP genes. | Seed storage protein modulation. | Nguyen et al. (2013) |
|  |  |  |  |

**Please refer to the main manuscript for reference details. For those not included in the main bibliography, please see below.**

Abdullah H, Rodriguez J, Salacup J, Castañeda IS, Schnell D, Pareek A, Dhankher O. Increased cuticle waxes by overexpression of WSD1 improves osmotic stress tolerance in Arabidopsis thaliana and Camelina sativa. Int J Mol Sci. 2021:22(10):5173. <https://doi.org/10.3390/ijms22105173>

Bengtsson J, Wallis J, Bai S, Browse J. The co-expression of two desaturases provides an optimized reduction of saturates in camelina oil. Plant Biotechnol J. 2023:21(3):497-505. <https://doi.org/10.1111/pbi.13966>

Choudhury R, Riesselman A, Pandey S. Constitutive or seed-specific overexpression of arabidopsis G-protein γ subunit 3 (AGG3) results in increased seed and oil production and improved stress tolerance in Camelina sativa. Plant Biotechnol J. 2014:12:49–59. <https://doi.org/10.1111/pbi.12115>

Duan Z, Ito K, Tominaga M. Heterologous transformation of Camelina sativa with high-speed chimeric myosin XI-2 promotes plant growth and leads to increased seed yield. Plant Biotechnol J. 2020:37:253–259. <https://doi.org/10.5511/plantbiotechnology.20.0225b>

Hu Z, Wu Q, Dalal J, Vasani N, Lopez H. Sederoff H, Qu R. Accumulation of medium-chain, saturated fatty acyl moieties in seed oils of transgenic Camelina sativa. PLoS ONE. 2017:12:e0172296. <https://doi.org/10.1371/journal.pone.0172296>

Huang A, Coutu C, Harrington M, Rozwadowski K, Hegedus D. Engineering a feedback inhibition-insensitive plant dihydrodipicolinate synthase to increase lysine content in Camelina sativa seeds. Transgenic Res. 2022:31(1):131-148. <https://doi.org/10.1007/s11248-021-00291-6>

Lee S, Kim H, Kim R, Suh M. Overexpression of Arabidopsis MYB96 confers drought resistance in Camelina sativa via cuticular wax accumulation. Plant Cell Rep. 2014:33:1535-1546. <https://doi.org/10.1007/s00299-014-1636-1>

Li N, Qi G, Sun X, Xu F, Wang D. Adhesion properties of camelina protein fractions isolated with different methods. Ind Crops Prod. 2015:69:263–272. <https://doi.org/10.1016/j.indcrop.2015.02.033>

Lyzenga WJ, Harrington M, Bekkaoui D, Wigness M, Hegedus DD, Rozwadowski KL. CRISPR/Cas9 editing of three CRUCIFERIN C homoeologues alters the seed protein profile in Camelina sativa. BMC Plant Biol. 2019:19(1):292. <https://doi.org/10.1186/s12870-019-1873-0>

Na G, Aryal N, Fatihi A, Kang J, Lu C. Seed-specific suppression of ADP-glucose pyrophosphorylase in Camelina sativa increases seed size and weight. Biotechnol Biofuels 2018:11:330. <https://doi.org/10.1186/s13068-018-1334-2>

Na G, Mu X, Grabowski P, Schmutz J, Lu C. Enhancing microRNA167A expression in seed decreases the α‐linolenic acid content and increases seed size in Camelina sativa. Plant J. 2019:98(2):346-358. <https://doi.org/10.1111/tpj.14223>

Okooboh G, Haferkamp I, Rühle T, Leister D, Neuhaus H. Expression of the plastocyanin gene PETE2 in Camelina sativa improves seed yield and salt tolerance. J Plant Physiol. 2023:290:154103. <https://doi.org/10.1016/j.jplph.2023.154103>

Park M, Choi H, Kim H. Physaria fendleri FAD3-1 overexpression increases ɑ-linolenic acid content in Camelina sativa seeds. Sci Rep. 2023:13(1):7143. <http://dx.doi.org/10.1038/s41598-023-34364-9>

Petrie J, Shrestha P, Belide S, Kennedy Y, Lester G, Liu Q, Divi U, Mulder R, Mansour M, Nichols P, et al. Metabolic engineering Camelina sativa with fish oil-like levels of DHA. PLoS ONE. 2014:9:e85061. <https://doi.org/10.1371/journal.pone.0085061>

Sarvas C, Puttick D, Forseille L, Cram D, Smith M. Ectopic expression of cDNAs from larkspur (Consolida ajacis) for increased synthesis of gondoic acid (cis-11 eicosenoic acid) and its positional redistribution in seed triacylglycerol of Camelina sativa. Planta. 2021:254(2):32. <https://doi.org/10.1007/s00425-021-03682-5>

Walsh D, Babiker E, Burke I, Hulbert S. Camelina mutants resistant to acetolactate synthase inhibitor herbicides. Mol Breeding. 2012:30:1053–1063. <https://doi.org/10.1007/s11032-011-9689-0>

Wang S, Blume R, Zhou Z, Lu S, Nazarenus T, Blume Y, Xie W, Cahoon E, Chen L, Guo L. Chromosome-level assembly and analysis of Camelina neglecta: a novel diploid model for Camelina biotechnology research. Biotechnol Biofuels Bioprod. 2024:17(1):17. <https://doi.org/10.1186/s13068-024-02466-9>

Willett W, Rockström J, Loken B, Springmann M, Lang T, Vermeulen S, Garnett T, Tilman D, DeClerck F et al Food in the Anthropocene: the EAT–Lancet Commission on healthy diets from sustainable food systems. The Lancet. 2019:393(10170):447 – 492

Yin Y, Raboanatahiry N, Chen K, Chen X, Tian T, Jia J, He H, He J, Guo Z, Yu L, et al. Class A lysophosphatidic acid acyltransferase 2 from Camelina sativa promotes very long-chain fatty acids accumulation in phospholipid and triacylglycerol. Plant J. 2022:112(5):1141-1158. <https://doi.org/10.1111/tpj.15999>

Zhang Y, Yu L, Yung K, Leung D, Sun F, Lim B. Over-Expression of AtPAP2 in Camelina sativa leads to faster plant growth and higher seed yield. Biotechnol Biofuels. 2012:5:19. <https://doi.org/10.1186/1754-6834-5-19>
